# Ophthalmic infections caused by *Aspergillus nidulans*: A case series and short review of literature 

**DOI:** 10.18502/cmm.7.4.8411

**Published:** 2021-12

**Authors:** Prachala G. Rathod, Atul Kumar, Radhika Tandon, Nishat H. Ahmed

**Affiliations:** 1 Microbiology Department, All India Institute of Medical Sciences, New Delhi, India; 2 Ophthalmology Department, All India Institute of Medical Sciences, New Delhi, India

**Keywords:** Antifungal susceptibility, *Aspergillus nidulans*, Ophthalmic infections

## Abstract

**Background and Purpose:**

Although *Aspergillus fumigatus* and *Aspergillus flavus* are more commonly implicated with ocular infections; there are some saprophytic species, such as *Aspergillus nidulans* (*A. nidulans*) which may occasionally lead to serious ocular infections. There is a paucity of data on ocular infections caused by *A. nidulans*. We report a case series of three ophthalmic infections caused by *A. nidulans* from a tertiary care eye center in North India.

**Case report:**

Three cases of ophthalmic infections, including two cases of keratitis and one case of recurrent endophthalmitis caused by *A. nidulans* were diagnosed at the ocular microbiology section of a tertiary eye care center. One case of keratitis had a history of ophthalmic surgery and underlying diabetes mellitus. The case of recurrent endophthalmitis had undergone cataract surgery in the recent past. Diminution of vision was the most common presenting feature in all three cases. The microbiological diagnosis was made by conventional microscopy and culture techniques.

**Conclusion:**

This case series illustrates the potential of uncommon fungal pathogens, such as *A. nidulans* to cause devastating ocular infections and has an emphasis on the importance of timely microbiological diagnosis in the management of such cases.

## Introduction

Fungal infections account for 25.6% to 36.7% of keratitis and 12% to 32% of endophthalmitis infections in India [ 1 - 3 ]. Vision-threatening ocular aspergillosis is not uncommon. Although *Aspergillus fumigatus* (*A. fumigatus*) and *Aspergillus flavus* (*A. flavus*) are the common *Aspergillus* spp. implicated with ocular infections, some saprophytic spp. have been reported to cause serious ocular infections, with the advent of invasive ocular procedures in recent years. Using the phylogenetic approach, the genus *Aspergillus* has been subdivided in six subgenera, 27 sections, and 75 series. *Aspergillus nidulans* (*A. nidulans*) is identified under the section *Nidulantes* [ 4 ].

*A. nidulans* has its niche in soil, water, and decaying vegetation. This saprophyte attracted the attention of the medical world only when it was recognized as an opportunistic pathogen causing invasive aspergillosis (IA) in chronic granulomatous disease patients [ 5 - 7 ]. *A. nidulans* has been described to cause such infections as osteomyelitis, cutaneous infections, endocarditis, and brain infections. However, it has rarely been implicated to cause ocular infections. 

This case series from a tertiary eye care center aimed to describe two cases of keratitis and one case of recurrent endophthalmitis caused by *A. nidulans*. To the best of our knowledge, the case of recurrent endophthalmitis caused by *A. nidulans* is the first of its kind to be reported from India.

## Case report

### Case no. 1

A 60-year-old female patient, known case of type-2 diabetes mellitus, began to experience pain and irritation in the left eye after undergoing a left penetrating keratoplasty. On examination, the left lid was edematous, and the corneal graft showed edema with buried sutures. An infiltrate of 2 x 1.5 mm and an epithelial defect was observed temporally. The visual acuity was limited to finger counting close to the face. Direct microscopy of a corneal scraping revealed septate hyphae, and fungal culture grew *A. nidulans*. The patient was prescribed natamycin 5% and voriconazole eye drop 1% along with oral voriconazole 200 mg for 14 days. However, due to minimal resolution of infection, loose sutures were sent to the microbiology lab for a culture that again grew *A. nidulans*. The infection resolved after two months with an extended voriconazole treatment, and there have been no recurrences to date.

### Case no. 2

A 65-year-old male farmer presented with diminution of vision and opacity in both eyes. He gave a history of redness and opacity in the right eye three years back and in the left eye one month back. The patient had been diagnosed with right anterior staphyloma and corneal melt in the left eye in a private hospital when he referred to our center. There was no history of ocular trauma, but a watery discharge and congestion were observed in both eyes. The visual acuity was limited to the perception of light in both eyes. The left eye examination revealed a 3x3 mm central corneal thinning. The case was diagnosed with left infective keratitis with corneal thinning and melts. The patient was empirically started on vancomycin 5%, tobramycin 1.3%, natamycin 5% eye drops, and oral ciprofloxacin for 14 days. On day 10 of presentation, the ulcer in the left eye had a 2.5 x 2.5 mm perforation, for which penetrating keratoplasty (PK) was performed, and an intraoperatively excised cornea was sent for microbiological workup. The direct microscopy did not demonstrate any bacterial and fungal etiology. Therefore, the patient was discharged with an empirical coverage of vancomycin 5%, natamycin 5%, tobramycin 1.3% eye drops, and oral ciprofloxacin for 7 days and a plan for weekly follow up visits. The fungal culture grew *A. nidulans* after 3 days. However, an oral antifungal could not be started as the patient did not visit the center again and was lost to follow-up.

### Case no. 3

A 59-year-old male presented with sudden onset of vision diminution, pain, and redness in the left eye after undergoing cataract surgery five days earlier. The visual acuity for distant vision in the left eye was hand movement close to the face. Ophthalmic examination showed conjunctival congestion and the presence of AC cells, exudates, and flare in the anterior chamber. A diagnosis of acute post-cataract surgery endophthalmitis was made. Subsequently, an Endophthalmitis Pars Plana Vitrectomy (PPV) with intravitreal antibiotic instillation was performed. Vitreous biopsy was sent for microbiological workup, and the patient was discharged with an empirical coverage of vancomycin 5%, tobramycin eye drops 1.3% and oral ciprofloxacin for one week. Fungal culture grew *A. nidulans*. However, the patient did not refer for his first follow-up, and an antifungal could not be prescribed. The patient visited the center 18 days later due to the deterioration of his condition, and the examination revealed a membrane on the left pupil along with a nasally located fungal ball. The patient was operated for PPV with intraocular lens and bag explants, vitreous lavage, and intravitreal voriconazole (100μg /0.1ml) instillation along with natamycin eye-drops 5%, and oral voriconazole 200 mg for one month. 

The patient’s symptoms did not mitigate with the given treatment, and a diagnosis of recurrent endophthalmitis was made. The PPV was repeated with intraocular lens and bag explants, accompanied with intravitreal amphotericin B (10μg /0.1ml) and vancomycin (1mg /0.1ml) instillation. The patient could not visit the center for follow-up due to the COVID-19 pandemic. On teleconsultation, the patient reported left eye discharge, for which he was advised to follow up physically.

**Figure 1:**
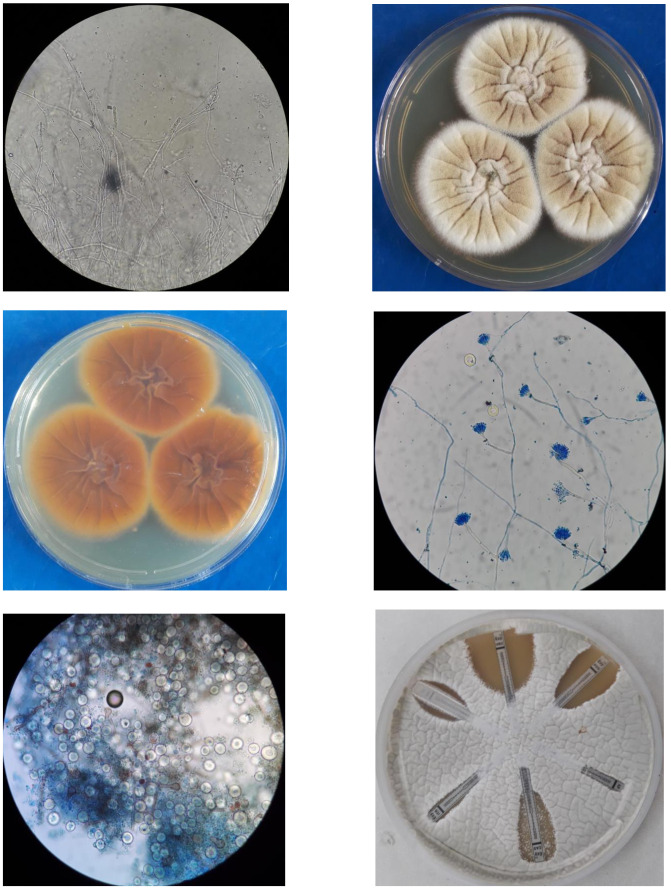
Morphological features of *Aspergilllus nidulans*: A) Potassium hydroxide mount of corneal specimen showing septate, hyaline hyphae, B) Fungal culture on Sabouraud Dextrose Agar (obverse), C) Fungal culture on Sabouraud Dextrose Agar (reverse), D) Lacto-phenol cotton blue mount showing biseriate vesicles on short conidiophores; E) Lacto-phenol cotton blue mount showing an open, round cleistothecium bearing red-brown lenticular ascospores; F) Lacto-phenol cotton blue mount showing numerous spherical, double walled ‘Hulle cells’

Samples from all three cases were received in the ocular microbiology section. The samples included a corneal scraping and loose sutures (from case no. 1); cornea excised in PK procedure (from case no. 2); and two vitreous biopsies (from case no. 3). All samples were processed for gram stain, potassium hydroxide (KOH) wet mount, and culture. Sheep Blood agar (5%) and Sabouraud Dextrose Agar (SDA) (HiMedia, Mumbai, India) were incubated at 37°C for 18 to 24 h and at 25°C for a minimum of 14 days. Primary microscopy of cases no. 1 and 3 showed thin branching septate hyphae with few pus cells (Figure1-A). Bacterial cultures were sterile in all three cases and SDA grew filamentous fungus between days 3 and 5. The isolation of fungi was considered significant if at least one of the following criteria was met: a) observation of fungal elements in primary microscopy and fungal growth in fungal culture medium (b) confluent fungal growth on the sample inoculation site [ 8 ]. The fungal growth from all samples was initially white cottony and later appeared glabrous. It matured with tan and smoky green alternative rings on the obverse and dark brown pigment on the reverse (Figure 1-B, C). Lactophenol cotton blue staining showed septate hyaline hyphae with short to medium length brown-tinged conidiophores. These conidiophores produced biseriate, flask-shaped vesicles covered by metulae and phialides on the upper part bearing round, smooth, green conidia in chains (Figure 1- D). After 10-14 days, large round cleistothecia were observed encompassing asci producing red-brown lenticular ascospores (Figure 1-E). The cleistothecia were surrounded by numerous large, spherical, thick, double-walled hyaline ‘Hulle Cells’ (Figure 1- F). The morphological characteristics of the isolates were confirmed through slide cultures of all the isolates. 

The E-strip method (HiMedia, Mumbai, India) was employed to determine the antifungal susceptibility pattern of all the isolates [ 9 ]. Minimum Inhibitory Concentration (MIC) was determined for amphotericin B, posaconazole, itraconazole, and voriconazole using E-test strip. All MIC values were recorded 24 and 48 h after the application of strips. The MIC was defined as the lowest concentration preventing any discernible growth. Table 1 presents the results of the antifungal susceptibility test of the three isolates. Clinical breakpoints (CBP) were not determined by the Clinical and Laboratory Standard Institute (CLSI) for Aspergillus species. Therefore, the results of antifungal susceptibility testing of amphotericin B, posaconazole, itraconazole, and voriconazole were interpreted following the ‘modes’ (most frequent MIC) documented for *Aspergillus* spp. in the M38 document by CLSI [ 10 ].

**Table 1:** Results of antifungal susceptibility testing of the three *Aspergillus* nidulans isolates

Case No	Amphotericin B (MIC in µg/ml)	Posaconazole (MIC in µg/ml)	Itraconazole (MIC in µg/ml)	Voriconazole (MIC in µg/ml)
Case 1	6	0.25	0.125	0.25
Case 2	24	0.19	0.19	0.19
Case 3	6	0.25	0.19	0.25

## Discussion

*Aspergillus* species are distributed widely in the environment and act as opportunistic pathogens in an immunocompromised group of patients. Infections, such as osteomyelitis, endocarditis, brain abscess, eumycetoma, and other cutaneous infections caused by *A. nidulans* have been reported worldwide [ 11 , 24 ]. Most of the human infections caused by *A. nidulans* are described in chronic granulomatous disease patients [ 5 - 7 ]. However, *A. nidulans* has also been identified as a pathogen in immunocompetent individuals [ 17 , 23 ].

Ophthalmic infections caused by *A. nidulans* are uncommon (Table 2). The reported cases include two cases of endophthalmitis and one case of contact lens infection. Among these endophthalmitis cases, one had a history of asthma followed by hospitalization and administration of intravenous corticosteroids along with a catheter insertion shortly before the occurrence of endophthalmitis. The authors speculated that catheter insertion might have been the possible source of infection [ 18 ].

**Table 2:** Compilation of case reports of ophthalmic infections caused by *Aspergillus nidulans*

Author	Year, country	Type of study	Type of infection	Diagnosis method	Treatment	Outcome
Mutulu FM *et al*. [15]	2016, Turkey	Case report	Endophthalmitis post cataract surgery	Culture	Voriconazole	Vision improved
Moret LM *et al*. [18]	2020, Spain	Case report	Endophthalmitis	PCR	Voriconazole	Vision improved
Lalitha C *et al*. [24]	2020, India	Case report	Contact lens infection	Culture	Natamycin	Resolution of infection

Increased successful outcomes and certain serious postoperative complications are boon and bane of advancements in diagnostic and management facilities for ocular disorders. Ocular surgeries may sometimes pave the way for the entry of environmental opportunistic fungal spores to inaccessible sites. Two out of three cases discussed in this case series had a history of previous ocular surgeries, and one post-operative case had underlying diabetes mellitus and was non-compliant with medications. No evident trigger or risk factor was identified in the third case.

Fungal endophthalmitis has been reported to be accounting for 18.6%-21.6% of all postoperative endophthalmitis cases in tropical countries like India [ 25 , 26 ]. Vitrectomy and intravitreal antimicrobial instillation remain the mainstay for the management of these cases. The Infectious Disease Society of America strongly recommends systemic and intravitreal administration of voriconazole for the treatment of *Aspergillus endophthalmitis* [ 27 ]. Vinekar A *et al*. [ 26 ] reported that multiple vitrectomies and intraocular lens (IOL) explantation along with careful removal of bag led to the resolution of infection. Nonetheless, case no. 3 in this case series did not have satisfactory results following multiple PPVs and IOL with bag explants. To the best of our knowledge, our case of recurrent fungal endophthalmitis caused by *A. nidulans* is the first case to be reported from India. 

Microbiological identification is essential and is the mainstay of diagnosing ocular fungal infections. Fungal culture is considered the gold standard method for diagnosing fungal infections, and molecular methods are recommended for the confirmation of rare isolates. However, the use of molecular methods is limited due to the lack of availability at all centers. It should be noted that all the three fungal isolates from our cases were diagnosed conventionally by KOH direct microscopy and culture on SDA medium due to lack of facility for molecular identification of fungal isolates at our center. The confirmation was carried out only by slide culture of the isolates, which can be regarded as the major limitation of the present case study.

Antifungal susceptibility of the fungal isolates guides the medical management in such refractory infections and contributes to the epidemiological data and resistance surveillance. Very few authors have reported antifungal susceptibility results of *A. nidulans* [ 9 ]. The comparison of the MIC values of amphotericin B with the mode MIC (ranging between 0.5-1mcg/ml) identified for *Aspergillus* species by CLSI showed high MIC values for all three isolates. In addition, all the three isolates showed MIC values within the mode range mentioned by CLSI (0.06-0.5 µg/ml) for triazoles (Table 1). Similar findings were noted by a few other authors [ 9 , 28 - 30 ]. Among the triazoles, voriconazole was reported to have broad-spectrum activity and be safe for both systemic and intravitreal instillation; therefore, it may be considered an alternative in cases whose infection is refractory to amphotericin B therapy.

## Conclusion

Ocular infections by common *Aspergillus* spp. are well documented. The authors reported ophthalmic infections caused by *A. nidulans*, a rare species of *Aspergillus*. High clinical suspicion is needed in such cases since timely diagnosis and prompt initiation of appropriate treatment are of paramount importance to salvage the precious vision of patients. Based on the results, azoles showed good activity against this pathogen and may play an important role in the medical management of such cases.

## Acknowledgement

The authors wish to thank the technical staff of the Ocular Microbiology section of the Dr. Rajendra Prasad Centre for Ophthalmic Sciences, All India Institute of Medical Sciences, New Delhi, India, for their technical help.

## Authors’ contribution

N.H.A. and P.G.R contributed to laboratory diagnosis and manuscript writing and editing.

A.K. and R.T. were involved in the clinical diagnosis of the patients.

## Conflict of Interest

The authors declare that they have no conflict of interest regarding the publication of the present study.

## Financial disclosure

The authors received no financial support for the current study.

## Ethical Considerations

The study was approved by the Ethics Committee of All India Institute of Medical Sciences, New Delhi, India (Ref: IEC-698/01.10.2021). Informed consent was obtained from all the patients.
